# Neutrophil Extracellular Traps Impair Intestinal Barrier Function during Experimental Colitis

**DOI:** 10.3390/biomedicines8080275

**Published:** 2020-08-05

**Authors:** Elliot Yi-Hsin Lin, Hsuan-Ju Lai, Yuan-Kai Cheng, Kai-Quan Leong, Li-Chieh Cheng, Yi-Chun Chou, Yu-Chun Peng, Yi-Hsuan Hsu, Hao-Sen Chiang

**Affiliations:** 1 Department of Life Science, National Taiwan University, Taipei 10617, Taiwan; elliot821012@gmail.com (E.Y.-H.L.); beta850703@gmail.com (H.-J.L.); b98b01048@ntu.edu.tw (Y.-K.C.); r08b21015@ntu.edu.tw (K.-Q.L.); jenny122439@gmail.com (L.-C.C.); z900215ro@gmail.com (Y.-C.P.); k450012@hotmail.com (Y.-H.H.); 2 Genome and Systems Biology Degree Program, National Taiwan University, Taipei 10617, Taiwan; joisechou0714@gmail.com

**Keywords:** neutrophil extracellular traps (NETs), intestinal barrier integrity, DNase I, DSS/TNBS-induced colitis

## Abstract

Aberrant neutrophil extracellular trap (NET) formation and the loss of barrier integrity in inflamed intestinal tissues have long been associated with inflammatory bowel disease (IBD). However, whether NETs alter intestinal epithelium permeability during colitis remains elusive. Here, we demonstrated that NETs promote the breakdown in intestinal barrier function for the pathogenesis of intestinal inflammation in mouse models of colitis. NETs were abundant in the colon of mice with colitis experimentally induced by dextran sulfate sodium (DSS) or 2,4,6-trinitrobenzene sulfonic acid (TNBS). Analysis of the intestinal barrier integrity revealed that NETs impaired gut permeability, enabling the initiation of luminal bacterial translocation and inflammation. Furthermore, NETs induced the apoptosis of epithelial cells and disrupted the integrity of tight junctions and adherens junctions. Intravenous administration of DNase I, an enzyme that dissolves the web-like DNA filaments of NETs, during colitis restored the mucosal barrier integrity which reduced the dissemination of luminal bacteria and attenuated intestinal inflammation in both DSS and TNBS models. We conclude that NETs serve a detrimental factor in the gut epithelial barrier function leading to the pathogenesis of mucosal inflammation during acute colitis.

## 1. Introduction

Inflammatory bowel diseases (IBD) comprise Crohn’s disease (CD) and ulcerative colitis (UC) that affect over 3.5 million people worldwide [1]. These diseases are characterized by aberrant immune responses against commensal microorganisms, leading to chronic intestinal inflammation [2]. In healthy individuals, the single-layered intestinal epithelium provides a barrier that physically and immunologically prevents direct contact between the luminal microbiota and the immune cells at the lamina propria [3]. However, genetic polymorphisms and environmental cues can both result in decreased gut epithelium integrity and increase intestinal permeability to innocuous microbiota. This predisposition eventually leads to abnormal innate and adaptive immune responses and underpins the pathogenesis of IBD [2,4].

Upon the initiation of the inflammatory response, circulating neutrophils transmigrate into the intestinal mucosa [5]. Although neutrophil migration into the inflamed tissues is an essential process of the immune defense, mucosal healing, and resolution of inflammation [6,7], increased neutrophil infiltration of the gut epithelium characterizes the histology of IBD, e.g., crypt abscesses, and is associated with endoscopic IBD severity [8]. Infiltrating neutrophils utilize several immune mechanisms, including phagocytosis, reactive oxygen species production, and degranulation, as well as the formation and release of a web-like structure called neutrophil extracellular trap (NET) [6,7]. NET is composed of decondensed chromatin filaments decorated with histones, neutrophil granules, and cytosolic proteins that locally defend the host against the penetrating microbes [9]. Although NET components play an antimicrobial role, excessive NET formation tends to induce a proinflammatory state and contributes to host cell injury [10]. Indeed, abundant NET-associated proteins and NET structures have been detected by proteomics and confocal microscopy analysis in intestinal biopsies from individuals with CD [11] and UC [12,13,14]. Furthermore, several NET-associated proteins, such as calprotectin and myeloperoxidase (MPO), are documented IBD biomarkers [15]. Furthermore, NETs amplify the inflammatory signals in UC and promote a hypercoagulable state in IBD, indicating their involvement in the pathogenesis of IBD [14,16]. However, the mechanisms by which NETs modulate intestinal barrier integrity and the progression of gut inflammation remain to be elucidated.

Here, we demonstrated that NET formation is enhanced in the inflamed intestinal mucosa of mice with experimental colitis, while inducing collateral damage in the surrounding tissue. NETs altered the integrity of tight junction and adherens junction proteins as well as promoted the intestinal cell death, leading to increased gut epithelium permeability and the translocation of commensal bacteria to distal tissues. By contrast, the digestion of NETs by a pharmacological intervention (DNase I treatment) in a mouse with intestinal inflammation restored the intestinal barrier integrity, resulting in the lower numbers of translocated luminal bacteria in the colon and mesenteric lymph nodes (MLNs), the reduced proinflammatory cytokine production, as well as the disease severity. Collectively, these findings indicate that NETs play a detrimental role in controlling the intestinal barrier function and initiation of experimental colitis.

## 2. Materials and Methods

### 2.1. Animals

Wild-type C57BL/6JNarl mice were obtained from National Laboratory Animal Center (NLAC) (NARLabs, Taipei, Taiwan). All mice were bred, housed in the animal facility at National Taiwan University (NTU) under specific pathogen-free (SPF) conditions, and maintained in a 12-h light–dark cycle with food and water ad libitum according to institutional guidelines. All experiments were conducted on female mice (7 weeks old) in accordance with the animal protocols approved by the Institutional Animal Care and Use Committee (IACUC) at NTU (approval # NTU-107-EL-109, 6 May 2018).

### 2.2. DSS-Induced Colitis and DNase I Administration

Seven-week-old female mice were randomly assigned into 4 groups and housed in 4 separate cages (1: Water+phosphate-buggered salin(PBS), 2: Water+DNase I, 3: dextran sulfate sodium (DSS)+PBS, 4: DSS+DNase I). Mice were fed 2.5% (wt/vol) DSS (molecular mass: 36,000-50,000 Da, MP Biomedicals, Santa Ana, CA, USA) in sterilized drinking water continuously for 8 d. The amount of DSS water consumed by each animal group was comparable. Control mice were given drinking water that did not contain DSS. In DNase I-administered groups, 250 U DNase I (BioShop Canada Inc., cat.# DRB001.100, Burlington, ON, Canada) were *i.v.* injected every other day on days 0, 2, 4, and 6, to deplete NET formation. PBS was *i.v.* injected as the vehicle control. Bodyweight and clinical manifestations (fecal bleeding and stool consistency) were examined every day of the experiment. The determination of clinical manifestations was scored as previously described [17]. On day 8, mice were euthanized by CO_2_, and their colons were removed and measured by ImageJ software.

### 2.3. TNBS-Induced Colitis and DNase I Administration

Trinitrobenzene sulfonic acid (TNBS)-induced acute colitis in mice was performed as previously reported [18]. Briefly, 150 µL of 1% (wt/vol) TNBS (Sigma-Aldrich, cat.# P2297, St. Louis, MO, USA) pre-sensitization solution was applied to the shaved skin area of anesthetized mice for pre-sensitization. Seven day post pre-sensitization, anesthetized mice were slowly intra-rectal (*i.r.*) administrated with 100 µL of 2.5% (wt/vol) TNBS solution (mixing 5 % (w/v) TNBS with equal volume of absolute ethanol) via a 3.5 F catheter. The mice were kept in head down position for 1 min to avoid TNBS solution excretion. In DNase I-administered groups, 250 U DNase I (BioShop Canada Inc., cat.# DRB001.100, Burlington, ON, Canada) were *i.v.* injected on days 0 and 2 to deplete NET formation. PBS was *i.v.* injected as the vehicle control. After 4 days, colon tissues were collected for further analysis.

### 2.4. Histopathological Analysis

For the DSS-induced colitis model, hematoxylin and eosin (H&E)-stained sections of colons were assessed by two blinded and professionally trained pathologists using a previously described scoring system [19]. Three independent parameters were determined: severity of inflammation (scores 0 to 3: none, slight, moderate, severe), depth of injury (scores 0 to 3: none, mucosal, mucosal and submucosal, transmural), and crypt damage (sores 0 to 4: none, basal 1/3 damaged, basal 2/3 damaged, only surface epithelium intact, entire crypt and epithelium lost). The score for each parameter was multiplied by a factor reflecting the percentage of tissue involvement (×1, 0% to 25%; ×2, 26% to 50%; ×3, 51% to 75%; ×4, 76% to 100%) and added. The maximum possible score was 40.

For the TNBS-induced colitis model, histological scoring was assessed using a previously described scoring system [18]. Scores 0 to 3: 0: none, 1: low level of inflammation, with scattered infiltrating mononuclear cells (1–2 foci), 2: moderate inflammation, with multiple foci, 3: high level of inflammation, with increased vascular density and marked wall thickening, 4: maximal severity of inflammation, with transmural leukocyte infiltration and loss of goblet cells.

### 2.5. ELISA

To prepare the extracts, freshly collected colonic or fecal samples were homogenized in PBS with 0.1% (vol/vol) Tween 20 (PBST) until clear supernatants were obtained. The level of mouse Il-1β, Tnf-α and Il-17a in colon extracts and lipocalin-2 levels in the stool were detected using Mouse IL-1 beta/IL-1F2 DuoSet ELISA kit (R&D systems, cat.# DY401-05), ELISA MAX™ Standard Set Mouse TNF-α (Biolegend, cat.#43092), ELISA MAX™ Standard Set Mouse IL-17A (Biolegend, cat.#432501), and Mouse Lipocalin-2/NGAL DuoSet ELISA kit (R&D systems, cat.# 1857), respectively, according to the manufacturer’s instructions.

To quantify NETs in mouse colonic homogenates, a capture-ELISA [20] for the detection of MPO associated with DNA was used, with modifications. Briefly, 96-well plates were coated with an anti-MPO antibody (R&D Systems, cat.# AF3667, 2 µg/mL) at 4 °C for 16 h. After washing three times with PBST, blocking buffer (PBS with 1% (wt/vol) bovine serum albumin) was added to each well and incubated for 1 h at room temperature. After discarding the blocking buffer, 10 µg total protein of colonic homogenates (100 µL) and 20 U DNase I (6.67 µL) were added to each well. After a 15-min incubation at room temperature, 1 µL of 0.5 M EDTA was added to each well to stop the DNase I reaction, and the mixtures were incubated at room temperature for additional 2 h. The wells were next washed with PBS and a peroxidase-labeled anti-DNA monoclonal antibody (Cell Death Detection ELISA, cat.# 1154467500: bottle 2, 1:40; Roche Diagnostics, Indianapolis, IN, USA) was added to the wells. The plate was incubated for 60 min at room temperature. After three washes with PBS, 100 µL of the peroxidase substrate 3,3′,5,5′-tetramethylbenzidine (Clinical Science Products, cat.# 01016-1-100, Mansfield, MA, USA) was added and the mixtures incubated 10 min at room temperature in the dark. Then, 100 µL of stop solution (2 N H_2_SO_4_) was added to each well and sample absorbance was measured at 450 nm by using an absorbance microplate reader (Molecular Devices, EMax Plus, San Jose, CA, USA).

### 2.6. Immunofluorescence Staining of NETs, Tight Junctions, and Adherens Junctions

Distal colon sections of mice were collected immediately after euthanizing and embedded in O.C.T (Tissue-Tek, cat.# 62550, Zoeterwoude, the Netherlands), cut into 8-µm serial sections (CM1900 cryostat, Leica, Wetzlar, Germany), and transferred to saline-coated slides. The sections were fixed in 4% paraformaldehyde for 15 min and then permeabilized with 0.1% (vol/vol) Triton X-100 for 10 min. The slides were blocked using 0.05% (vol/vol) Tween-20 in PBS (PBST) containing 10% (vol/vol) serum (GIBCO, cat.#160000044, Waltham, MA, USA) from the same species as the secondary antibodies and incubated for 1 h at room temperature. NET-related proteins were detected using antibodies against citH3 (1:200, Abcam, cat.# ab5103, Cambridge, MA, USA), MPO (1:200, R&D Systems, cat.# AF3667, Minneapolis, MN, USA), and NE (1:200, Abcam, cat.# ab21595), by incubating at 4 °C overnight. Tight junctions or adherens junctions were detected with antibodies against occludin (Invitrogen, cat.# 33-1500, 1:200), ZO-1 (Thermo Fisher, cat.# 61-7300, 1:200), E-cadherin (Cell Signaling, cat.#3195S, 1:200) for 4 °C overnight. After washing with PBS for 15 min, Alexa Fluor 594 donkey anti-rabbit IgG (H+L) (1:500, Life Technologies, cat.# A21207, Carlsbad, CA, USA), Alexa Fluor 488 donkey anti-goat IgG (H+L) (1:500, Life Technologies, cat.# A11055), or Alexa Fluor 594 goat anti-mouse IgG (H+L) (1:500, Life Technologies, cat.# A11032) were added as secondary antibodies, and incubated for 1 h at room temperature. Hoechst 33,342 (1:1000, Invitrogen, cat.# H3570, Carlsbad, CA, USA) was used as the nucleic acid dye. Samples were incubated with the dye for 5 min at room temperature, and then rinsed two times with PBS. The slides were mounted in 20 µL (per coverslip) of fluorescent mounting medium (Dako, cat.# S3023, Santa Clara, CA, USA). Images were acquired by using a Zeiss microscope (Zeiss, Ovserver.Z1, Oberkochen, Germany) equipped with a 3-megapixel camera (Zeiss, Axiocam 503 mono, Oberkochen, Germany). NET counts were calculated based on the co-localization of MPO, citH3, and DNA per 200X high-power field (HPF). At least 3 different fields were count per mouse.

### 2.7. FACS Analysis

To obtain colonic epithelium and lamina propria cells, entire colons were flushed with PBS to empty the luminal contents, opened longitudinally and cut laterally into 0.5cm pieces. Such pieces were shaken at 200 rpm for 10 min at 37 °C in Ca^2+^/Mg^2+^-free HBSS with 5% FBS, 5 mM EDTA, 10 mM HEPES, and 0.15% DTT. To obtain colonic epithelium cell suspensions (IE), the cell suspension was filtered on a 100 µm cell strainer and stained for fluorescence activated cell sorting (FACS). To obtain single lamina propria cell suspensions (LP), the remaining pieces were minced and digested for 20 min at 37 °C in HBSS medium containing 10% FBS, 2 mg/mL collagenase IV (Sigma) and 0.05 mg/mL DNase I (Roche). The cell suspension was filtered on a 40 µm cell strainer and stained for FACS.

1 × 10^6^ colonic lamina propria cells were washed in PBS supplemented with 3% FBS (PBS/FBS). Cells were first incubated with 2.4G2 Mouse Fc block (BD Pharmingen, San Diego, CA, USA) in PBS with 10% FBS for 20 min at 4 °C. The cells were then washed and stained with fluorescent-conjugated antibodies for 30 min at 4 °C. The following antibodies were purchased from BD Pharmingen and used for our analysis: Ly6G-PE, CD11b-APC, and F4/80-AF488. Fluorescently labeled cells were acquired on a FACSCanto II flow cytometer (BD Biosciences, San Diego, CA, USA) and analyzed using FlowJo Analysis Software (Tree Star, Inc., Ashland, OR, USA).

### 2.8. Gene Expression Analysis

Colonic tissues were washed carefully before the extraction of RNA to remove residual feces. RNA was extracted using the 1-bromo-3-choropropane (Sigma-Aldrich, cat.# B9673, St. Louis, MO, USA) and the phenol:chloroform:iso-amyl alcohol (25:24:1, VWR, cat.# VWRV0883, Radnor, PA, USA) extraction method. The same amount of RNA (2000 ng per sample) was reverse-transcribed to cDNA by using the High-Capacity cDNA Reverse-Transcription kit (Applied Biosystems, cat.# 4368814, Foster City, CA, USA) by following the manufacturer’s instructions. For the reaction, 100 ng of cDNA was mixed with Fast SYBR Green Master Mix (Applied Biosystems, cat.# 4385612) and analyzed using the Applied Biosystems QuantStudio Real-Time PCR system. The cDNA amplification was performed as follows: denaturation for 10 s at 95 °C and annealing for 30 s at 60 °C, with 40 cycles of denaturing and annealing. Primer sequence was listed in Supplementary Table S1. Gene expression was calculated by using the ΔΔ*Ct* method. Each sample was assayed in duplicate. Values were normalized to the expression of *Tbp* since its expression is stable in mice with DSS colitis [21]. For the quantification of fecal bacterial 16S rDNA, fecal genomic DNA was extracted from the fresh collected feces with a QIAamp DNA stool mini kit (Qiagen, cat.#51604). A total of 250 pg DNA from each mouse fecal sample was used as a template in each PCR reaction. The primer sequences used in this study are listed in the Supplemental Table S1.

### 2.9. Intestinal Permeability Assay

The assay was performed as previously described [22]. Briefly, mice were water fasted overnight before the test. Each mouse was given an oral gavage of 150 µL of 10 mg/mL fluorescein isothiocyanate (FITC)-dextran (Sigma-Aldrich, cat.# FD4) 4 h before euthanizing, and transferred to a new cage without food and bedding while fasting, with only water provided. Before the FITC-dextran gavage, pre-test plasma samples were collected for subsequent normalization of the readings for each mouse, to compare the relative fluorescence of FITC-dextran in circulation. Four hours after FITC-dextran gavage, mice were euthanized using a carbon dioxide chamber, and blood was directly collected from the heart. The collected blood was mixed with an acid-citrate-dextrose solution (Sigma-Aldrich, cat.# C3821, 15% (vol/vol)) by inversion. The blood samples were centrifuged (5000 rpm, 10 min, at 4 °C), the plasma was diluted 1:10 in PBS, and 100 µL of the diluted samples was loaded per well of 96-well plates. Fluorescence was detected at 530 nm after excitation at 485 nm by using a fluorescence microplate reader (Molecular Devices, FilterMax F3).

### 2.10. Bacterial Translocation Assay

The colon and MLNs were aseptically collected from euthanized mice on day 8. The tissues were homogenized in sterile PBS. Tissue homogenates were serial diluted and spread on Luria–Bertani agar plates. After incubation at 37 °C for 24 h, colony-forming units (CFUs) were counted. The numbers are presented as CFU/g of tissue.

### 2.11. TUNEL Assay

Apoptosis was detected by using the DeadEnd Fluorometric TUNEL system (Promega, cat.# G3250, Madison, WI), in accordance with the manufacturer’s protocol. The colon tissues treated with clean water or DSS in the presence or absence of DNase I were removed, rinsed with PBS, fixed in 10% formaldehyde, and embedded in paraffin. The tissues were counterstained with Hoechst 33,342 (Invitrogen, cat.#H3570, Carlsbad, CA, USA). Apoptotic cells were visualized under a fluorescence microscope (Zeiss, Ovserver.Z1, Oberkochen, Germany). ImageJ software was used for image analysis and signal quantification.

### 2.12. Statistical Analysis

The data are presented as the mean ± SEM. Differences were analyzed by using an unpaired, two-tailed *t*-test (two-group comparisons) or one-way ANOVA followed by Turkey’s multiple comparison (comparisons of three or more groups) by using GraphPad Prism version 7.0a (GraphPad, La Jolla, CA, USA). *p*-values less than or equal to 0.05 were considered statistically significant and were denoted as *, *p* < 0.05; **, *p* < 0.01; ***, *p* < 0.001; ****, *p* < 0.0001.

## 3. Results

### 3.1. NET Formation is Enhanced in the Colon of Mouse with Dextran Sulfate Sodium (DSS)-Induced Colitis

**Figure 1:**
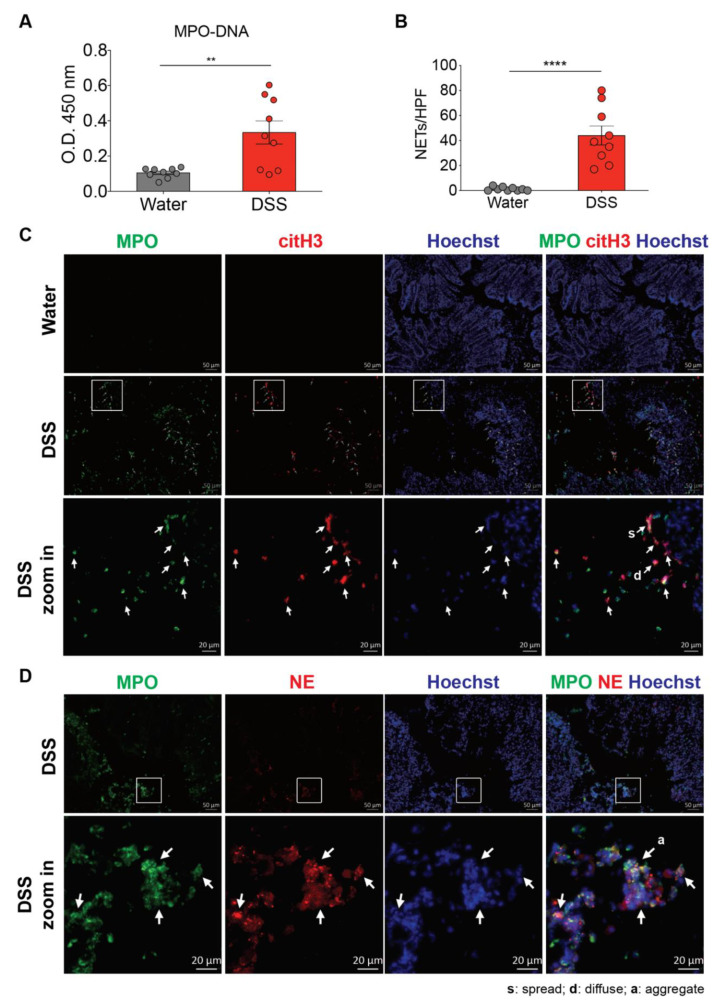
Neutrophil extracellular traps (NETs) are induced in the colon of dextran sulfate sodium (DSS)-treated mice. (**A**) NET release was measured in colon homogenates of wild-type C57BL/6 mice 8 d after the consumption of clean water or 2.5% DSS, by using myeloperoxidase (MPO)-DNA ELISA. (The results are pooled data from two separate experiments. *n* = 9 mice per group.) (**B**,**C**) NET formation counts per 200X high-power field (HPF) and representative NET release (white arrows) in the colon of wild-type C57BL/6 mice 8 d after the consumption of clean water or 2.5% DSS, assessed by immunofluorescence microscopy of MPO (green), citrullinated histone H3 (citH3; red), and Hoechst 33342-stained DNA (blue). Bottom row, enlargement of the area outlined in the second-row images. (The results are pooled data from two separate experiments. *n* = 9 mice per group.) (**D**) Representative NET release (gray arrows) in the colon of wild-type C57BL/6 mice 8 d after the consumption of 2.5% DSS, assessed by immunofluorescence microscopy of MPO (green), neutrophil elastase (NE; red), and Hoechst 33342-stained DNA (blue). Bottom row, enlargement of the area outlined in the second-row images (*n* = 9 mice per group). s: NETs in spread form, d: NETs in diffuse form, a: aggregate NET. The data represent the mean ± SEM. Statistical analyses were performed using unpaired, two-tailed *t*-test. ** *p* < 0.01, **** *p* < 0.0001. Scale bars, 50 µm. 20 µm in zoom in pictures.

Since neutrophils are the first immune cells recruited to the colon during colitis, we reasoned that the infiltrating neutrophils are activated, which would lead to NET formation [14]. To determine whether NET production is increased in the colon during colitis, we utilized a mouse model of DSS-induced colitis, which shares many similarities with human UC [23]. Although we did not detect the presence of MPO-DNA complexes in the plasma that was reported in mice received 3.5% DSS for 6 d (Supplemental Figure S1) [24], colonic homogenates of mice with 2.5% DSS-induced colitis contained significantly more MPO-DNA complexes than colonic homogenates of the control mice (Figure 1A). We next characterized NETs in the colon sections of the control and DSS mice. We used an immunofluorescence approach that allowed visualization of the co-localization of extracellular DNA, MPO (a neutrophil marker), and nuclear citrullinated histone H3 (citH3, NET marker) or neutrophil elastase (NE). DSS treatment induced a robust neutrophil infiltration and histone H3 citrullination in the mouse colon (Figure 1B,C). The presence of the NET structures was not restricted to the lamina propria and epithelium, and NETs were also present in the intestinal lumen. Furthermore, in the mouse model, colonic neutrophils had changes in nuclear size and shape during NET formation. We observed neutrophils with either diffuse or spread DNA staining co-localized with MPO and citH3/NE, reflecting the early stages of NET formation that their chromatin had not spread out and late stages of NET formation that neutrophils already fully spread NETs, respectively [25], in the intestine of DSS-induced mice. Furthermore, we also detected NETs between neutrophils at the sites of neutrophil aggregation (Figure 1D). By contrast, no NETs were apparent in the colon of control mice. Together, these findings suggest that the DSS treatment robustly induces NET formation in mice during an acute phase of intestinal inflammation.

### 3.2. Degradation of NETs During Intestinal Inflammation Suppresses the Progression of Experimental Colitis in Mouse

**Figure 2:**
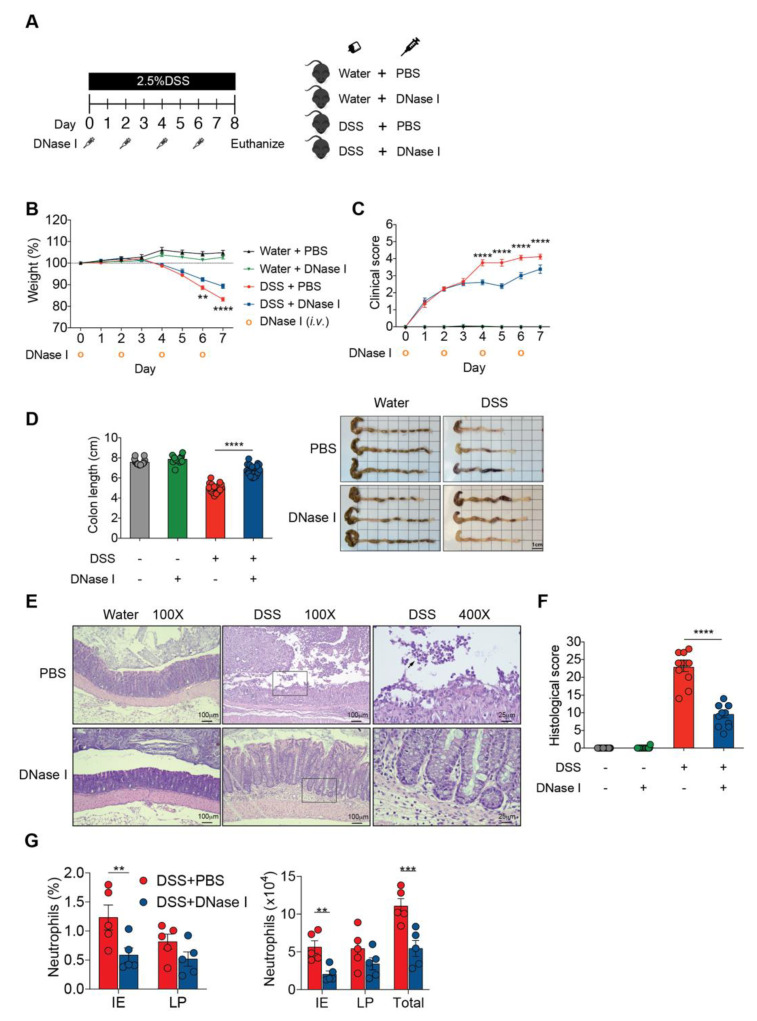
DNase I treatment attenuates DSS-induced colitis in mice. (**A**) Schematic overview of the experiment. Colitis was induced by providing 2.5% DSS in the drinking water for 8 d. Clean water was fed to the controls. Phosphate-buffered saline (PBS) or 250 U/dose DNase I was intravenously (*i.v.*) administered to wild-type C57BL/6 mice every other day, on days 0, 2, 4, and 6. (**B**,**C**) Daily weight and total clinical scores of the control or DSS-treated mice after PBS or DNase I treatment. (**D**) Colon lengths and representative colon images of the control or DSS-treated mice with or without DNase I treatment. (**B**–**D**) The results are pooled data from four separate experiments. *n* = 15 mice per control groups and *n* = 18 mice per DSS groups. (**E**) Representative hematoxylin and eosin (H&E) staining of the colon, 400X high-power field (HPF) images are the enlargement of the area outlined in the 100X HPF images. The black arrow indicates neutrophils in the lumen. (**F**) Histopathology score of the control or DSS-treated mice with or without DNase I treatment. (**E,F**) The results are pooled data from two separate experiments. *n* = 9 mice per control groups and *n* = 12 mice per DSS groups. (**G**) Percentage and absolute numbers of neutrophils (CD11b ^+^ Ly6G^+^F4/80^-^) in the intestinal epithelium (IE) and lamina propria (LP) of the colon (Total). *n* = 5 mice per group. The data represent the mean ± SEM. Statistical analyses were performed using (**B**–**D**,**F**), one-way ANOVA with Turkey’s multiple comparison or (**G**), an unpaired two-tailed *t*-test. ** *p* < 0.01, *** *p* < 0.001, **** *p* < 0.0001. Scale bar, 1 cm in (**D**), 100 µm in 100× pictures and 25 µm in 400× pictures of (**E**).

Based on the above observations, demonstrating a pronounced NET formation in the colon of mice with DSS-induced colitis, we then evaluated the in vivo contribution of NETs to the initiation and progression of intestinal inflammation. We intravenously (*i.v.*) administered exogenous DNase I every second day during DSS administration to degrade NETs in mice with DSS-induced colitis, and monitored the progression of gut inflammation (Figure 2A). Mice receiving DSS and DNase I exhibited reduced weight loss (Figure 2B) and a lower disease activity index (improved stool consistency, reduced fecal occult blood, and rectal bleeding) than phosphate-buffered saline (PBS)-treated DSS mice (Figure 2C). Administration of DNase I to mice with experimental colitis also led to diminished colon length shrinkage, a signature phenotype of DSS-induced colitis [23], compared with the controls (Figure 2D). Histological analysis further indicated that the DNase I treatment protected DSS mice from loss of surface epithelium with underlying inflammation (Figure 2E,F). Indeed, flow cytometry analysis of the colon tissues suggested that a significantly reduced and a moderately decreased number of neutrophils (CD11b^+^Ly6G^+^F4/80^-^ cells) were recruited to the epithelium (IE) and the lamina propria (LP) of colon of DNase I-treated DSS mice, respectively (Figure 2G and Supplemental Figure S2).

**Figure 3:**
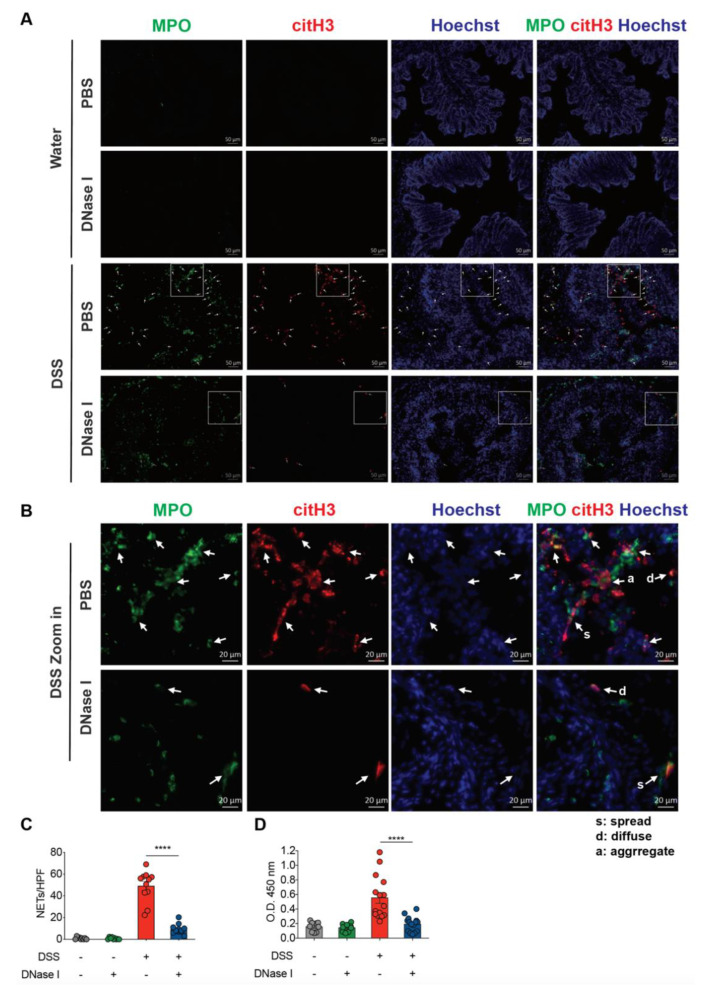
DNase I treatment significantly degrades NET structure in the colon of mice fed DSS. (**A**) Representative NET release (white arrows) in the colon of PBS control or DNase I-treated mice 8 d after the consumption of clean water or 2.5% DSS, assessed by the immunofluorescence microscopy of myeloperoxidase (MPO; green), citrullinated histone H3 (citH3; red), and Hoechst 33342-stained DNA (blue). (**B**) Enlargement of the area outlined in (**A**). White arrows indicate NETs. s: NETs in spread form, d: NETs in diffuse form, a: aggregate NET. (**C**) NET formation counts per 200X high-power field (HPF) in each group. (**A**–**C**) The results are pooled data from two separate experiments. *n* = 9 mice in water control groups and *n* = 12 mice in DSS-treated groups, at least 3 images/mouse. (**D**) NET release, measured in colon homogenates of PBS- or DNase I-administered wild-type C57BL/6 mice 8 d after the consumption of clean water or 2.5% DSS, by using MPO-DNA complex ELISA. The results are pooled data from three separate experiments. *n* = 9 mice per control groups and *n* = 15 mice per DSS groups. The data represent the mean ± SEM. Statistical analyses were performed using one-way ANOVA with Turkey’s multiple comparison. **** *p* < 0.0001. Scale bars, 50 µm in (**A**). 20 µm in (**B**).

To better characterize and confirm that the above phenotype was associated with a significantly reduced NET formation in the colon of DSS mice administered DNase I compared with the PBS-treated group, we again employed immunofluorescence staining to investigate the co-localization of DNA, MPO, and citH3 in the intestinal tissue sections, and ELISA to observe the colonic MPO-DNA complex formation (Figure 3). Administration of DNase I to the control mice did not induce neutrophil recruitment or histone H3 citrullination in the mouse colon, suggesting that exogenous DNase I had a minimal effect on NET formation in non-DSS mice in vivo (Figure 3A–C). By contrast, DNase I treatment significantly decreased the presence of MPO-DNA complexes in the colon homogenates of mice with DSS-induced colitis (Figure 3D). Furthermore, the pattern of diffuse (d), spread (s), and aggregate (a) DNA staining co-localizing with MPO and citH3 staining that resembled NETs in the colon sections was strongly diminished but not entirely abolished in DSS mice treated with DNase I (Figure 3A,B). Together, these observations highlight the impact of NET formation on the pathogenesis of colitis in vivo.

### 3.3. Disruption of NETs in Mouse Ameliorates the Intestinal Inflammation During DSS-Induced Colitis

**Figure 4:**
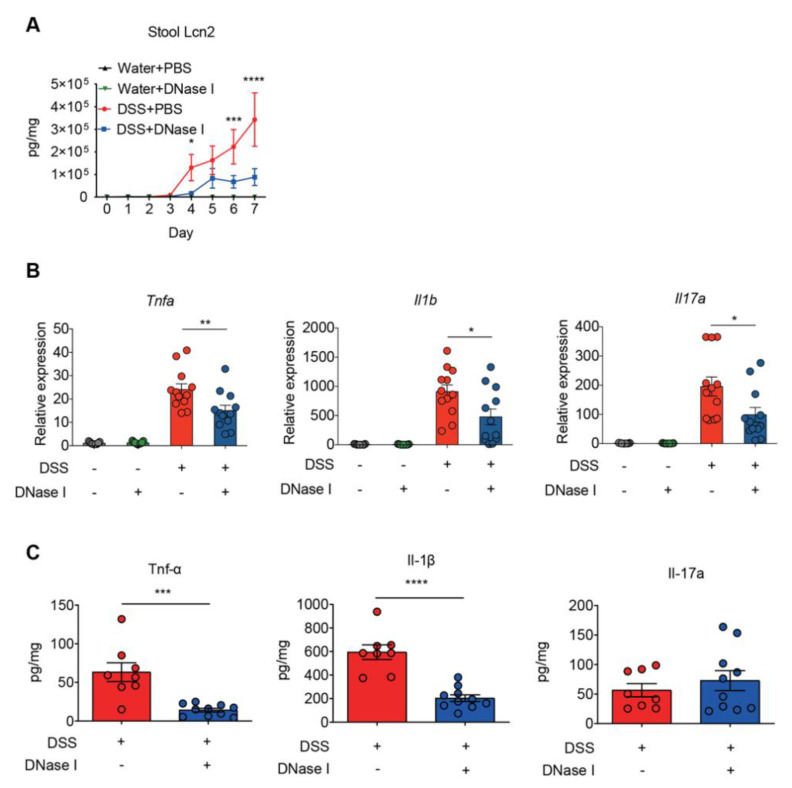
Disruption of NETs reduces the colonic inflammation in mouse fed DSS-containing water. (**A**) Fecal lipocalin-2 (Lcn2) levels. Fecal samples obtained from PBS- or DNase I-administered wild-type C57BL/6 mice 8 d after the consumption of clean water or 2.5% DSS were analyzed at different time points by ELISA. The results are pooled data from two separate experiments. *n* = 9 mice per control groups and *n* = 12 mice per DSS groups. (**B**) Quantitative RT-PCR analysis of *Tnfa*, *Il1b*, and *Il17a* mRNA levels in the colon of control and DSS mice treated with PBS or DNase I. Values are normalized to the expression of *Tbp*. The results are pooled data from two separate experiments. *n* = 9 mice per control groups and *n* = 12 mice per DSS groups. (**C**) Levels of Tnf-α, Il-1β, and Il-17a were determined in the colon homogenates of PBS- or DNase I-administered wild-type C57BL/6 mice 8 d after the consumption of 2.5% DSS. The results are pooled data from two separate experiments. *n* = 9 mice per PBS group and *n* = 10 mice per DNase I group. The data represent the mean ± SEM. Statistical analyses were performed using one-way ANOVA with Turkey’s multiple comparison. * *p* < 0.05, ** *p* < 0.01, *** *p* < 0.001, **** *p* < 0.0001.

To further examine whether DNase I treatment influenced the inflammatory and immune signatures of colitis in the control and DSS-induced mice, we measured the daily fecal levels of lipocalin-2, a sensitive biomarker for intestinal inflammation [26], and the expression of proinflammatory cytokines in the colon tissue of animals at the end of DSS treatment. As shown in Figure 4A, the levels of fecal lipocalin-2 were significantly increased on day 4 and continued to increase further in a time-dependent manner in all groups of mice given DSS. Intriguingly, DNase I treatment abolished such a time-dependent increase in DSS-fed mice. Furthermore, the expression of colonic proinflammatory cytokines, such as *Tnfa*, *Il1b*, and *Il17a*, in DSS mice treated with DNase I was reduced when compared with the PBS-treated group (Figure 4B). Likewise, the colon homogenates of DNase I-treated DSS mice contained less Tnf-α and IL-1β than PBS-treated DSS mice (Figure 4C). Collectively, these observations suggest that the administration of DNase I limits intestinal inflammation and thus protects the mouse against DSS-induced colitis.

### 3.4. NETs Alter Intestinal Permeability and Barrier Integrity in a Mouse Model of DSS-Induced Colitis

**Figure 5:**
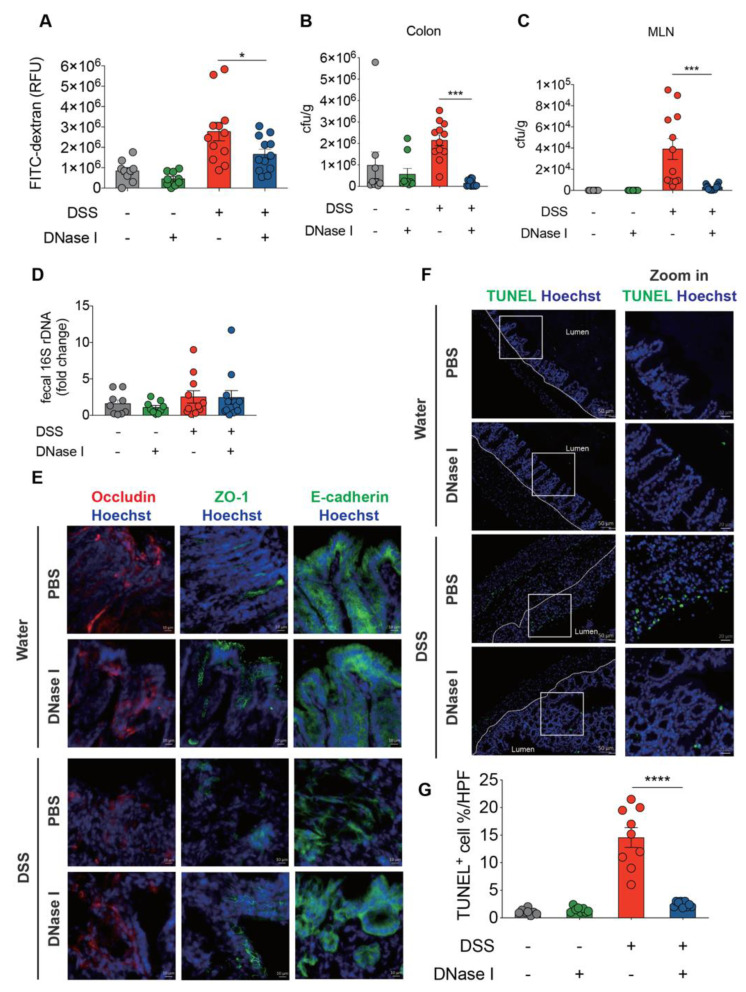
NETs alter intestinal barrier function and cause the apoptosis of intestinal cells in the colon of DSS-treated mice. (**A**) Intestinal permeability was determined by quantifying the amount of fluorescein isothiocyanate (FITC)-dextran levels in the serum 4 h after its oral gavage. PBS- or DNase I-administered wild-type C57BL/6 mice fed clean water or 2.5% DSS were tested on day 8 from the beginning of the DSS treatment. (**B**,**C**) Bacterial counts in the colon and mesenteric lymph nodes (MLN) of control or DSS mice treated with PBS or DNase I were determined on day 8. (**D**) Quantitative PCR analysis of relative amount of 16S rDNA in the feces of control and DSS mice treated with PBS or DNase I on day 8. (A-D) The results are pooled data from two separate experiments. *n* = 9 mice per control groups and *n* = 12 mice per DSS groups. (**E**,**F**) Representative immunofluorescence staining of occludin, ZO-1, E-Cadherin, and Hoechst 33342-stained DNA and representative fluorescent images of terminal deoxynucleotidyl transferase dUTP nick-end labeling (TUNEL, green) staining of the colon tissues isolated from PBS- or DNase I-administered wild-type C57BL/6 mice on day 8 after the consumption of clean water or 2.5% DSS. Slides were counterstained with Hoechst 33,342 (blue). (**G**) Percentage of apoptotic cells per 200X high-power field (HPF) in each group listed in f. The results are pooled data from two separate experiments. *n* = 8 mice per control groups and *n* = 9 mice per DSS groups. The data represent the mean ± SEM. Statistical analyses were performed using A-D, G one-way ANOVA with Turkey’s multiple comparison or E, unpaired two-tailed *t*-test. * *p* < 0.05, *** *p* < 0.001, **** *p* < 0.0001. Scale bar, 10 µm in E; 50 µm in the left panel and 20 µm in the zoom of F.

The above findings suggest that excessive NET release and deposition in the colon exacerbate the pathogenesis of colitis. Since a breakdown in the epithelial barrier function in DSS-induced mice would facilitate the mucosal invasion of luminal microorganisms, we next asked whether NET is an important modulator of intestinal epithelial barrier integrity during colitis in vivo. PBS- or DNase I-treated C57BL/6 mice were given 2.5% DSS in drinking water for 8 d, and the intestinal barrier permeability was then assayed using a fluorescein isothiocyanate (FITC)-labeled dextran. As expected, the intestinal barrier permeability in DSS mice apparently increased compared to the control mice (Figure 5A). By contrast, administration of DNase I, which dissolved the NET structure in vivo, resulted in a significantly reduced recovery of FITC-dextran from the serum of DSS-treated mice. These observations suggest that NETs profoundly enhance the deterioration of intestinal barrier integrity after DSS-induced injury. Disruption of epithelial barrier function in the colon causes bacterial translocation to different tissues [27]. We therefore asked whether NETs were also responsible for the luminal bacterial invasion/translocation in DSS mice. To assess the bacterial translocation to distal tissues, the numbers of colony-forming units (CFUs) in the colon and mesenteric lymph nodes (MLNs) were determined on day 8 in the model shown in Figure 2A. Significantly less bacteria were recovered from the colon of DNase I-treated DSS mice compared with PBS-administered DSS mice (Figure 5B). Moreover, we also observed a profound reduction of CFUs in the MLNs and cecum of DSS mice treated with DNase I (Figure 5C and Supplemental Figure S3A). These decreases were not likely caused by variations in luminal bacteria since the amount of 16s rDNA in the feces of DNase I-treated and PBS-administered DSS mice was comparable (Figure 5D). Overall, these observations established that DSS-induced NET formation contributes to increased bacterial translocation by altering the intestinal permeability and barrier function.

Since increased intestinal permeability may reflect a differential expression of epithelium structural proteins, we next asked whether the expression of the tight junction complex, occludin and ZO-1 as well as the adherens junction protein E-cadherin was altered in the colon of DNase I-treated DSS mice. As determined by immunofluorescence staining and quantitative RT-PCR, the expression levels of tight junction protein occludin and ZO-1 were significantly increased in DSS mice that received DNase I compared with PBS-treated controls (Figure 5E and Supplementary Figure S3B). Taken together, these observations suggest that dissociation of NET structure by DNase I was correlated with the restoration of tight junction complex, occludin and ZO-1, and adherens junction component E-cadherin in colitic mouse.

### 3.5. DSS-Induced NETs Promote Apoptosis of Intestinal Cells In Vivo

Aberrant NET formation causes collateral tissue damage in vivo [28]. Accordingly, we therefore hypothesized that the decreased barrier function in DSS-induced colitis could be explained by the induction of intestinal epithelial cell apoptosis by NETs, to facilitate the translocation of luminal microbes across the colon [29]. In this context, we anticipated that the administration of DNase I in colitic mouse would reduce the extent of apoptosis of epithelial cells. To test this, we performed a terminal deoxynucleotidyl transferase dUTP nick-end labeling (TUNEL) assay. Indeed, the number of TUNEL-positive cells in the colonic tissues in DNase I-treated DSS mice was profoundly decreased compared to that in PBS-treated DSS controls 8 d after colitis induction (Figure 5F,G). Furthermore, TUNEL staining of colonic tissue sections of DNase I-treated control mice revealed a limited number of apoptotic intestinal cells, indicating that DNase I administration did not induce intestinal cell death in non-DSS mice that show minimum NET formation and intact intestinal integrity. Overall, these findings confirmed that the presence of NETs in the colon causes collateral damage to the intestinal cells during DSS-induced colitis.

### 3.6. DNase I Administration Restores Intestinal Integrity and Decreases Intestinal Inflammation in Mice with TNBS-Induced Colitis

**Figure 6:**
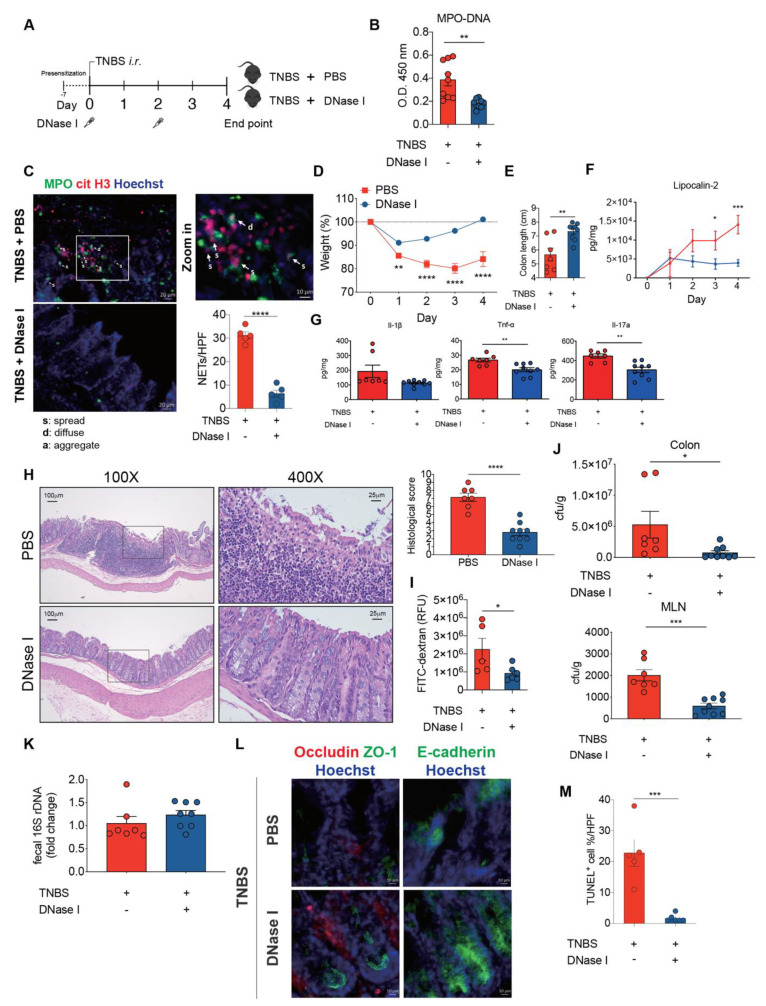
DNase I administration reduces intestinal inflammation and restores intestinal barrier function in mice with 2,4,6-Trinitrobenzenesulfonic acid (TNBS)-induced colitis. (**A**) Schematic overview of the experiment. Colitis was induced by the intra-rectal (*i.r.*) injection of TNBS into pre-sensitized C57BL/6 mice. PBS or 250 U/dose DNase I was *i.v.* administered to mice on day 0 and 2. (**B**) NET release, measured in colon homogenates of PBS- or DNase I-administered TNBS-induced colitis mice on day 4, by using MPO-DNA complex ELISA. *n* = 9 mice per group. (**C**) Representative NET release (white arrows) in the colon of PBS control or DNase I-treated mice 4 d after TNBS administration, assessed by immunofluorescence microscopy of myeloperoxidase (MPO; green), citrullinated histone H3 (citH3; red), and Hoechst 33342-stained DNA (blue). *n* = 5 mice per PBS group and *n* = 5 mice per DNase I group. (**D**) Daily weight of TNBS mice after PBS or DNase I treatment. (**E**) Colon lengths of TNBS-treated mice with or without DNase I treatment. (**F**) Fecal lipocalin-2 levels were analyzed from fecal samples obtained from PBS- or DNase I-administered TNBS mice at different time points by ELISA. (**G**) Protein level of Il-1β, Tnf-α, and Il-17A were determined in the colon homogenates of PBS- or DNase I-administered TNBS mice on day 4 by ELISA. (**H**) Representative H&E staining of the colon and histopathology score of the TNBS mice with or without DNase I treatment. 400× images are the enlargement of the area outlined in the 100X images. (D-H) The results are pooled data from two separate experiments. *n* = 7 mice per PBS group and *n* = 9 mice per DNase I group. (**I**) Intestinal permeability was determined by quantifying the amount of FITC-dextran levels in the serum 4 h after its oral gavage. PBS- or DNase I-administered TNBS mice were tested on day 4. *n* = 5 mice per PBS group and *n* = 6 mice per DNase I group. (**J**) Bacterial counts in the colon and MLN of TNBS mice treated with PBS or DNase I were determined on day 4. (**K**) Quantitative PCR analysis of relative amount of 16S rDNA in the feces of TNBS mice treated with PBS or DNase I on day 4. h. J and K results are pooled data from two separate experiments. *n* = 7 mice per PBS group and *n* = 8 or 9 mice per DNase I group. (**L**) Representative immunofluorescence staining of occludin, ZO-1, E-Cadherin, and Hoechst 33342-stained DNA of the colon tissues isolated from PBS- or DNase I-administered TNBS mice on day 4. (**M**) Percentage of apoptotic cells per 200× high-power field (HPF) in the colon of TNBS mice with or without DNase I treatment. *n* = 5 mice per PBS group and *n* = 6 mice per DNase I group. The data represent the mean ± SEM. Statistical analyses were performed using unpaired two-tailed *t*-test. * *p* < 0.05, ** *p* < 0.01, *** *p* < 0.001, **** *p* < 0.0001. Scale bar, 20 µm in c, 100 µm in 100× pictures and 25 µm in 400× pictures of H, 10 µm in L.

To verify that the protective effects of DNase I administration can be recapitulated on another mouse colitis model, we utilized the acute 2,4,6-trinitrobenzene sulfonic acid (TNBS) colitis model (Figure 6A) that is mainly mediated by hapten-induced T cell responses [30] with signatures of abundant neutrophil infiltration in the colon [31]. TNBS mice that had received DNase I administration exhibited the decreased presence of MPO-DNA complexes in the colon homogenates and fluorescence staining of DNA, MPO, and citH3 co-localization in the intestinal tissue sections (Figure 6B,C). These DNase I-treated TNBS mice also exhibited reduced weight loss, diminished colon length shrinkage, decreased levels of fecal lipocalin-2 and colonic proinflammatory cytokine expression, as well as better histopathological scores, when compared to TNBS mice treated with PBS (Figure 6D—H). Moreover, DNase I treatment significantly reduced the recovery of FITC-dextran from the serum of TNBS mice (Figure 6I). DNase I treatment further suppressed the luminal bacterial dissemination into the colon and MLN of TNBS-treated mice (Figure 6J). Similarly, DNase I treatment had a minimal effect on the number of luminal bacteria since the amount of 16s rDNA in the feces of DNase I-treated and PBS-administered TNBS mice was comparable (Figure 6K). The reduced bacterial translocation into the MLN was most likely due to the restoration of tight junctions and adherens junctions (Figure 6L) and reduced TUNEL^+^ cells (Figure 6M) in the colon of TNBS mice with DNase I administration. In summary, our data again suggest that the disruption of NET structure by DNase I ameliorates intestinal inflammation and restores gut barrier integrity and function in mice with TNBS colitis.

## 4. Discussion

**Figure 7:**
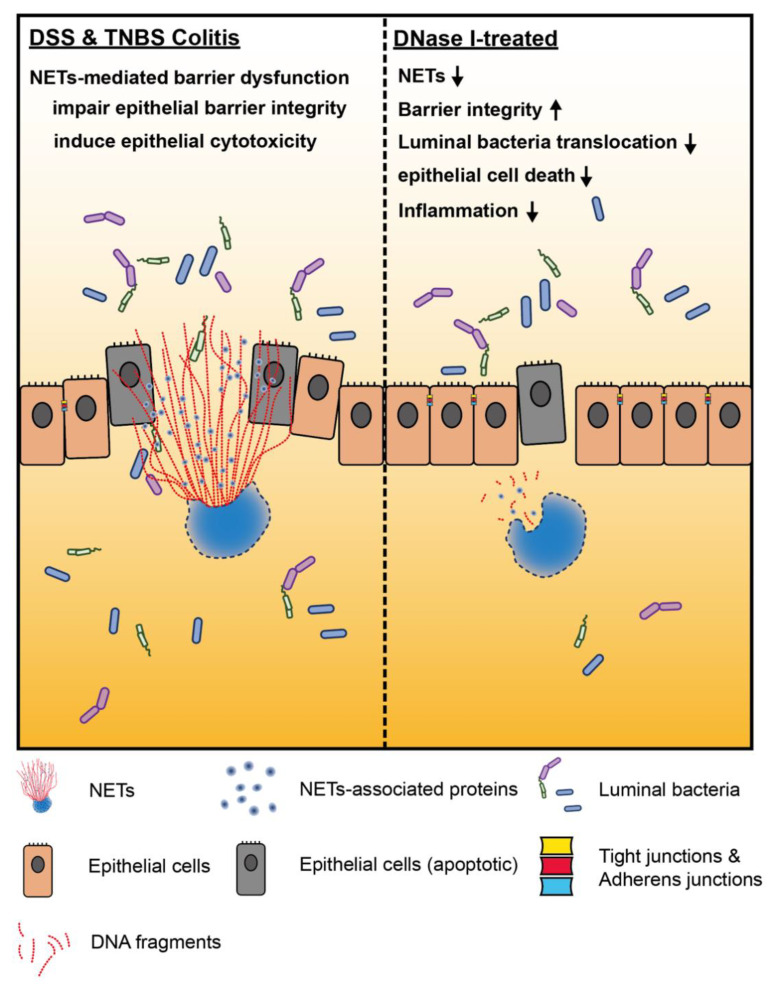
Neutrophil extracellular traps (NETs) exacerbate mouse experimental colitis by impairing intestinal barrier function. (Left) NETs are abundant in the colon of mouse in DSS-induced or TNBS-induced colitis models. In the colon of non-treated mouse, aberrant NET formation promotes the apoptosis of intestinal cells during colitis. NETs also alter intestinal epithelial permeability leading to luminal bacterial translocation into the colon and MLN as well as gut inflammation in vivo. Mechanistically, histones are the major protein components of NET structure that decrease the intestinal barrier integrity and function, as well as promotes the cytotoxicity of intestinal epithelial cells. (Right) Disruption of NET structure with DNase I in mice with DSS-induced or TNBS-induced colitis protects the host from intestinal inflammation and injury by restoring the intestinal barrier integrity and function that prevent luminal bacterial translocation into the colon and MLN, suggesting that NETs play a distinct role that is required for the development and pathogenesis of colitis.

In the current study, we aimed to evaluate the effect of NETs on intestinal homeostasis and the progression of gut inflammation during colitis. We demonstrated that NETs are abundant in the colon of mouse with experimental DSS and TNBS colitis. We also showed that NET formation impairs the intestinal epithelial permeability and function, leading to an increased translocation of luminal bacteria in vivo. The altered gut barrier integrity in the DSS and TNBS mouse was most likely associated with reduced expression of adherens and tight junction proteins and the apoptosis of epithelium, as collateral damage of NET release in the mouse colon. Remarkably, the disruption of NET structure by DNase I in DSS and TNBS mouse models protected the host against intestinal inflammation and injury, increased gut barrier function, as well as restricted luminal bacteria dissemination, suggesting that NETs have a distinct function required for the development and pathogenesis of colitis (Figure 7).

Neutrophils are among the first immune cells recruited to the site of inflammation [7]. The extent and numbers of infiltrating neutrophils in the intestinal mucosa of patients with IBD positively correlate with the presence of active CD and UC [32,33]. In this context, the neutrophils may employ several antimicrobial mechanisms, such as phagocytosis, degranulation, or NETs, to control the penetrating luminal microbes [34]. Of note, neutrophils isolated from IBD patients are more prone to NET formation than those from healthy controls [14,16]. Accordingly, an elevated abundance of neutrophil- and NET-associated components has been recently shown in both CD [11] and UC [12,13,14] colonic biopsies. In the animal model used in the current and other studies [14,24], DSS treatment induces and recapitulates NET generation in the mouse colon. We further demonstrate that TNBS treatment also promotes NET formation in the inflamed mouse colon. Although the detection of NET formation in the intestine remains challenging, as the NET formation process is dynamic, by using immunofluorescence staining of frozen colonic sections in the current study, we successfully captured the co-localization of three NET-associated molecules in DSS and TNBS mice (MPO, NE, and citH3) with the DNA backbone at the lamina propria, colonic epithelium, and the intestinal lumen. We also observed in the inflamed colon, neutrophils vary in nuclear size and shape for NET formation. While some neutrophils already fully released NETs (spread form), other neutrophils had the decondensed nuclei colocalized with MPO and citH3 or NE (diffuse form) and yet released NET structure. This dynamic and continuous process of NET formation in the colon during colitis is consistent with previous ex vivo findings [35,36]. Such NET signature was further supported herein by the ELISA of MPO-DNA complexes, detecting NETs in the colon homogenates.

Since their discovery in 2005, the putative role of NETs in immunity has been exemplified by their antimicrobial activity. Because the majority of NET-associated proteins are also critical for phagocytosis and degranulation [6,7], we originally assumed that the dissociation of NETs by DNase I during colitis would negatively impact the clearance of mucosal invasion by the commensal bacteria in the colon. Surprisingly, we found the opposite. The observed reduction of bacterial CFUs in the colon of DSS and TNBS mice upon DNase I treatment is probably associated with an as yet unknown mechanism compensating for the loss of specific NET contribution to the immune defense against the invading luminal microbes. This could be explained by a recent study showing that degradation of NETs by DNase I facilitates neutrophil antimicrobial killing against pneumococcus through the increased phagocytosis and MPO activity [37]. DNase I-mediated NET degradation would also release the functional antimicrobial peptides that are no longer attached to DNA backbone to boost antimicrobial activity [37] without affecting neutrophil viability [38]. Furthermore, we also demonstrated that DNase I treatment restored the intestinal barrier function during colitis. Consequently, fewer luminal bacteria can disseminate across the mucosa.

Except for the well-documented innate antimicrobial responses of NETs, increasing evidence suggests that NETs are a putative source of proinflammatory molecules that may promote atherosclerosis [39], diabetes [40], cystic fibrosis [41], sepsis [42], systemic lupus erythematosus [43], and thrombosis [44]. NETs have also been linked to intestinal damage in a rat model of lipopolysaccharide-induced sepsis [45]. The administration of a pan-peptidyl arginine deiminase (PAD) inhibitor Cl-amidine [46] or streptonigrin [14], a selective peptidyl arginine deiminase-4 (PAD4) inhibitor that blocks histone citrullination for NET formation, reduces the clinical symptoms of DSS-induced colitis. Moreover, circulating NETs induce the prothrombotic state of colitis [24]. Apart from these published findings, data presented in the current study further highlight the destructive effect of NETs on the intestinal permeability and barrier function in the pathogenesis of colitis and its behind mechanism. Therefore, the invading luminal bacteria may benefit from the collateral damage of mucosal integrity by NETs that leads to worsening intestinal inflammation.

Although the mechanisms that dismantle NET in vivo are still poorly understood, it has been shown that serum endonuclease DNase I produced and released by the pancreas is essential for NET degradation [36]. Furthermore, DNase I facilitates the engulfment of NETs by macrophages [47]. Intriguingly, the serum DNase I activity in patients with IBD is profoundly lower than that in healthy individuals [48]. Together, these observations further highlight the notion that the lack of NET degradation has probably deleterious consequences in IBD. Experiments presented in the current study demonstrated that DNase I treatment ameliorates experimental mouse colitis via a timely removal of NETs in the colon and restoration of intestinal integrity. We speculate that DNase I treatment would detangle the DNA backbone and dislodge NET-associated proteins to minimize the cytotoxic effects of histones. It is also worth mentioning that an intravital imaging-based study has shown that, although DNase I completely degrades DNA, it fails to remove NET-associated proteins, including histones from the endothelial wall and thus is less protective against systematic inflammation [49]. It would be of interest to examine whether NETs-associated proteins were still attached to the mucosa even after the removal of DNA backbone by DNase I. These findings may explain why we sometimes observed the residual inflammation in the colon of DSS mice received DNase I treatment.

In conclusion, the presented findings underscore the collateral damage caused by NETs to the mucosal surfaces during intestinal inflammation. NETs therefore play an unintended adverse role in the development of colitis that must be considered for treatment. Since the intestine is a dynamic environment with continuous interactions between the gut mucosa and commensal microorganisms, deliberate inhibition of NET formation in patients with IBD without understanding the specific contribution of NETs to inflammation in the gut in more detail would be precarious. It is possible that the small benefits of eliminating NETs to restore the intestinal barrier integrity would be outweighed by unwanted effects, e.g., increased microbial penetration and systematic inflammation associated with the loss of NET-dependent and/or NET-initiated antimicrobial defense against the invading microbes. Clarifying the underlying mechanism by which NETs contribute to defective mucosal barrier and intestinal inflammation could lead to a more detailed understanding of neutrophil biology in the initiation and pathogenesis of human IBD.

## Supplementary Materials

The following are available online at https://www.mdpi.com/2227-9059/8/8/275/s1, Figure S1: NET release was not detected in the plasma of DSS-induced colitis model in C57BL/6 mouse. Colitis was induced by supplying 2.5% DSS in drinking water for 8 d. Mice that received water without DSS served as the controls (n = 6 mice per group). NET release was measured by using MPO-DNA ELISA.; Figure S2: Flow cytometric analysis of intestinal epithelium cells (IE) or lamina propria cells (LP) isolated from the colon of DSS-induced colitis with PBS or DNase I treatment on day 8. Representative plots showing cells were initially gated on CD11b+ cells followed by staining of antibodies against Ly6G and F4/80. CD11b+Ly6G+F4/80- cells were defined as neutrophils; Figure S3: NETs alter intestinal barrier function and gene expression in the colon of DSS-treated mouse. (A) Bacterial count in the cecum of control or DSS mice treated with PBS or DNase I were determined on day 8. Results are pooled data from three separate experiments. n = 12 mice per control groups and n = 12 mice per DSS groups. (B) Quantitative RT-PCR analysis of occluding (Ocln) and ZO-1 (Tjp1) mRNA levels in the colon of control and DSS mice treated with PBS or DNase I. Values are normalized to the expression of Tbp. Results are pooled data from two separate experiments. n = 9 mice per control groups and n = 12 mice per DSS groups; Table S1: qPCR primers used in this study.
