# Supplementary material for: Neutrophil Extracellular Traps Impair Intestinal Barrier Function during Experimental Colitis
Source: Biomedicines. 2020 Aug 5;8(8):275. doi: 10.3390/biomedicines8080275 (PMC7459452; doi:10.3390/biomedicines8080275)
Supplement: Supplementary file 1 [file biomedicines-08-00275-s001.pdf]

Neutrophil extracellular traps impair intestinal barrier function during experimental colitis

Supplemental Document

Elliot Yi-Hsin Lin[1], Hsuan-Ju Lai[1], Yuan-Kai Cheng[1], Kai-Quan Leong[1], Li-Chieh Cheng[1], Yi-Chun Chou[2], Yu-Chun Peng[1], Yi-Hsuan Hsu[1], and Hao-Sen Chiang[1,2*]

[1]Department of Life Science, National Taiwan University, Taipei, Taiwan

[2]Genome and Systems Biology Degree Program, National Taiwan University, Taipei, Taiwan

[*]Corresponding author:

Hao-Sen Chiang, Ph.D.

R818 Life Science Building

1 Sec 4 Roosevelt Road

Taipei 10617, Taiwan

Phone: +886-2-3366-2454

Fax: +886-2-2363-6837

E-mail: hschiang@ntu.edu.tw

Gene name	Primer sequences
mouse *Il1b*	Forward primer: 5’-TCA CAG CAG CAC ATC AAC AA-3’<br>Reverse primer: 5’-TGT CCT CAT CCT GGA AGG TC-3’
mouse *Tnfa*	Forward primer: 5’-TAG CCA GGA GGG AGA ACA GA-3’<br>Reverse primer: 5’-TTT TCT GGA GGG AGA TGT GG-3’
mouse *Il17a*	Forward primer: 5’-CAG GAC GCG CAA ACA TGA-3’<br>Reverse primer: 5’-GCA ACA GCA TCA GAG ACA CAG AT-3’
mouse *Ocln*	Forward primer: 5’-TGG CAA GCG ATC ATA CCC AG-3’<br>Reverse primer: 5’-CCT CTT GCC CTT TCC TGC TT-3’
mouse *Cldn1*	Forward primer: 5’-GAT GTG GAT GGC TGT CAT TG-3’<br>Reverse primer: 5’-CCT GGC CAA ATT CAT ACC TG-3’
mouse *Tjp1*	Forward primer:5’-ACC CGA AAC TGA TGC TGT GGA TAG-3’<br>Reverse primer: 5’-AAA TGG CCG GGC AGA ACT TGT GTA-3’
mouse *Tbp*	Forward primer: 5’-ACC GTG AAT CTT GGC TGT AAA C-3’<br>Reverse primer: 5’-GCA GCA AAT CGC TTG GGA TTA-3’
bacterial 16S rDNA	Forward primer: 5’-CCA TGA AGT CGG AAT CGC TAG-3’<br>Reverse primer: 5’-ACT CCC ATG GTG TGA CGG-3’

Supplemental Table 1. qPCR primers used in this study.

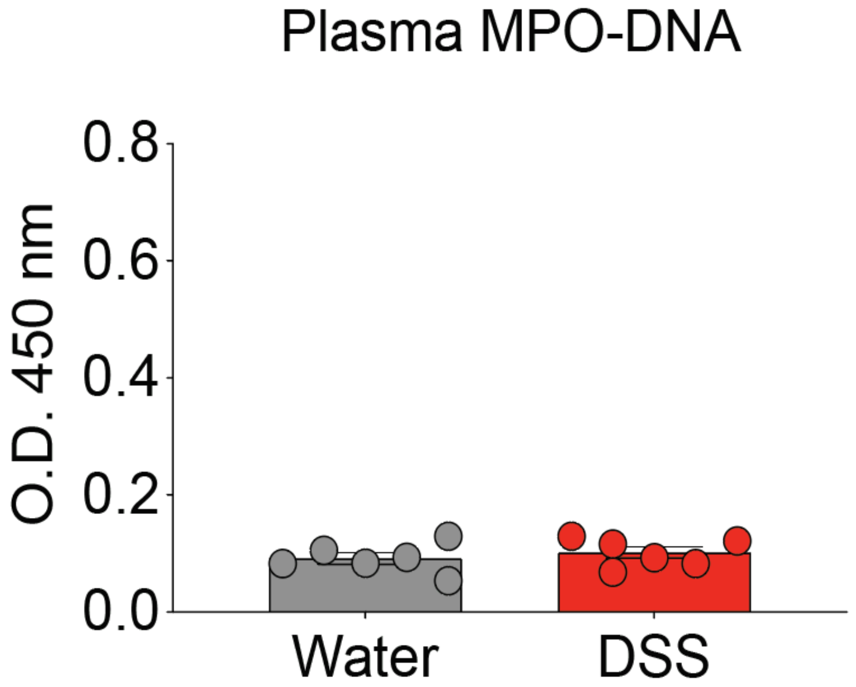

Supplemental Figure 1. NET release was not detected in the plasma of DSS-induced colitis model in C57BL/6 mouse. Colitis was induced by supplying 2.5% DSS in drinking water for 8 d. Mice that received water without DSS served as the controls (*n*=6 mice per group). NET release was measured by using MPO-DNA ELISA.

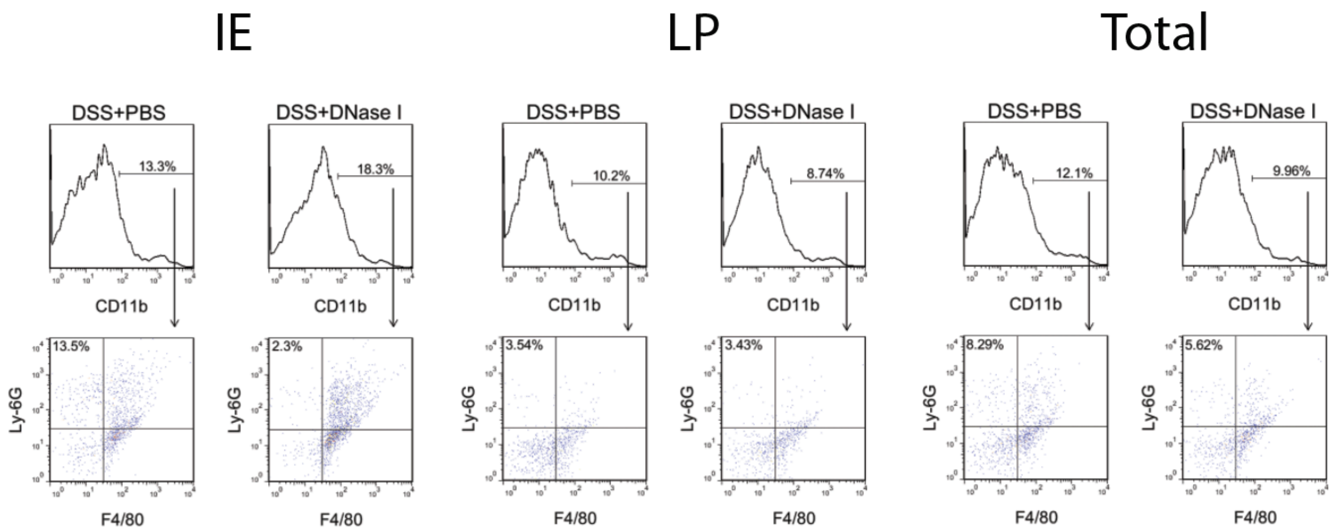

Supplemental Figure 2. Flow cytometric analysis of intestinal epithelium cells (IE) or lamina propria cells (LP) isolated from the colon of DSS-induced colitis with PBS or DNase I treatment on day 8. Representative plots showing cells were initially gated on $CD11b^+$ cells followed by staining of antibodies against Ly6G and F4/80. $CD11b^+Ly6G^+F4/80^-$ cells were defined as neutrophils.

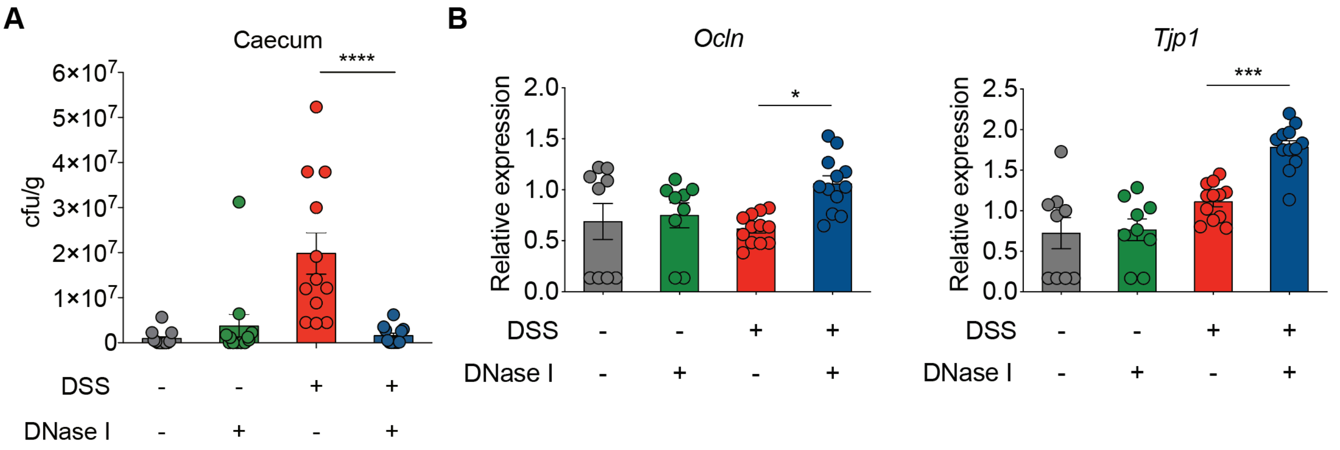

Supplemental Figure 3. NETs alter intestinal barrier function and gene expression in the colon of DSS-treated mouse. (**A**) Bacterial count in the cecum of control or DSS mice treated with PBS or DNase I were determined on day 8. Results are pooled data from three separate experiments. *n*=12 mice per control groups and *n*=12 mice per DSS groups. (**B**) Quantitative RT-PCR analysis of occluding (*Ocln)* and ZO-1 *(Tjp1)* mRNA levels in the colon of control and DSS mice treated with PBS or DNase I. Values are normalized to the expression of *Tbp*. Results are pooled data from two separate experiments. *n*=9 mice per control groups and *n*=12 mice per DSS groups.
